# Radiation exposure of adrenal vein sampling: a German Multicenter Study

**DOI:** 10.1530/EJE-18-0328

**Published:** 2018-07-31

**Authors:** C T Fuss, M Treitl, N Rayes, P Podrabsky, W K Fenske, D A Heinrich, M Reincke, T-O Petersen, M Fassnacht, M Quinkler, R Kickuth, S Hahner

**Affiliations:** 1 Department of Medicine I, Division of Endocrinology and Diabetology, University Hospital, University of Würzburg, Würzburg, Germany; 2 Department of Clinical Radiology, Ludwig-Maximilians-University, Munich, Germany; 3 Department of General, Visceral and Transplant Surgery, University Hospital Leipzig, Leipzig, Germany; 4 Department of Radiology, Charité Campus Virchow Klinikum, Berlin, Germany; 5 Leipzig University Medical Center, Integrated Center for Research and Treatment Adiposity Diseases, Leipzig, Germany; 6 Internal Medicine (Endocrinology and Nephrology), University of Leipzig, Leipzig, Germany; 7 Department of Endocrinology, Ludwig-Maximilians-University, Munich, Germany; 8 Department of Diagnostic and Interventional Radiology, University Hospital Leipzig, Leipzig, Germany; 9 Endokrinologie in Charlottenburg, Endokrinologie Praxis am Stuttgarter Platz, Berlin, Germany; 10 Department of Radiology, University Hospital Würzburg, Würzburg, Germany

## Abstract

**Objective:**

Adrenal vein sampling (AVS) represents the current diagnostic standard for subtype differentiation in primary aldosteronism (PA). However, AVS has its drawbacks. It is invasive, expensive, requires an experienced interventional radiologist and comes with radiation exposure. However, exact radiation exposure of patients undergoing AVS has never been examined.

**Design and methods:**

We retrospectively analyzed radiation exposure of 656 AVS performed between 1999 and 2017 at four university hospitals. The primary outcomes were dose area product (DAP) and fluoroscopy time (FT). Consecutively the effective dose (ED) was approximately calculated.

**Results:**

Median DAP was found to be 32.5 Gy*cm^2^ (0.3–3181) and FT 18 min (0.3–184). The calculated ED was 6.4 mSv (0.1–636). Remarkably, values between participating centers highly varied: Median DAP ranged from 16 to 147 Gy*cm^2^, FT from 16 to 27 min, and ED from 3.2 to 29 mSv. As main reason for this variation, differences regarding AVS protocols between centers could be identified, such as number of sampling locations, frames per second and the use of digital subtraction angiographies.

**Conclusion:**

This first systematic assessment of radiation exposure in AVS not only shows fairly high values for patients, but also states notable differences among the centers. Thus, we not only recommend taking into account the risk of radiation exposure, when referring patients to undergo AVS, but also to establish improved standard operating procedures to prevent unnecessary radiation exposure.

## Introduction

Primary aldosteronism (PA) is the most common curable cause of secondary hypertension and characterized by the autonomous adrenocortical oversecretion of aldosterone leading to cardiovascular and metabolic complications (1, 2, 3, 4, 5). Most commonly, the aldosterone excess either results from bilateral adrenal hyperplasia (BAH) or from a unilateral aldosterone-producing adenoma (APA) (6). Correct distinction between unilateral or bilateral disease is of high importance due to different therapeutic approaches: whereas PA due to APA may be cured by adrenalectomy, patients with BAH receive life-long treatment with mineralocorticoid receptor antagonists (7). AVS is currently recommended as standard diagnostic tool for subtype differentiation in PA (2). However, AVS has its drawbacks: It is an invasive and expensive procedure with poor standardization regarding sampling protocols and interpretation of results among centers (8, 9, 10). Success rates and complications such as adrenal hemorrhage also differ depending on the experience of the interventionalist (11, 12). Furthermore, there is not only an ongoing debate regarding the use of AVS itself (13, 14, 15), but also its superiority in comparison to adrenal CT scan in terms of clinical outcome (9, 16). However, the SPARTACUS-trial raised controversial discussions itself due to its study design and patient selection (15, 17). The clinical usefulness of more recent approaches to replace AVS by molecular imaging is yet to be defined within clinical trials (18, 19).

Another argument brought forward against AVS is the potentially high radiation dose, to which patients are submitted during the procedure. Even though there are proven risks linked to radiation exposure, e.g. an increased incidence of cancer, to our knowledge, there is no available data so far addressing this particular problem. We, therefore, aimed to retrospectively analyze radiation exposure of AVS in four different specialized centers across Germany.

## Patients and methods

### Patients

We performed a retrospective analysis to assess radiation exposure caused by AVS. In all patients, informed consent for performance of AVS within clinical routine had been obtained in accordance with respective local regulations. The analysis was approved by the Ethics Committee of the University of Würzburg (AZ121/17). Overall, 658 AVS performed between 1999 and 2017 in Berlin (center 1, *n* = 53), Leipzig (center 2, *n* = 52), Munich (center 3, *n* = 400) and Würzburg (center 4, *n* = 151) with documented information regarding dose area product (DAP, Gy*cm^2^), fluoroscopy time (FT, min) and, if available, performing radiologist and success of cannulation based on cortisol measurements were included in the analysis, without specific exclusion criteria. We furthermore collected information on sampling locations and imaging modalities for each center. Data were obtained at each participating center and sent to the coordinating center after anonymization.

### Assessment of radiation exposure

Assessed radiation measures were FT (min), DAP (Gy*cm^2^) and effective dose (ED, mSv). FT constitutes for the amount of time during the procedure, in which fluoroscopy is used. It poorly correlates with other dose indicators. DAP is defined as the product of the radiation dose to air multiplied by the area of X-ray field, as measured by an ionization chamber mounted on the X-ray collimator. DAP is used to assess the radiation exposure of irrigated tissues and forms the basis for calculation of ED. ED represents the stochastic risk related to ionizing radiation and was approximately estimated as previously described: ED (mSv) = DAP (Gy*cm²) × 0.2 (mSv/Gy*cm²) (20).

Across participating sites the following angiography systems were used: Artis Zee, Axiom Artis XA C, Axiom Artis BA, Multistar TOP, Polystar XA (each Siemens Healthcare), Innova 4100 (GE Healthcare) and ALLURA Xper FD System (Philips Healthcare). For details, see Table 1.

**Table 1:** Overview of imaging modalities.

Center	Angiography system	Sampling locations number (location)	DSA number (location)	Frames per second
1	ALLURA Xper FD System	6 (LAV, RAV, 2xLRV, 2xVCI)	3* (2xRAV, LAV)	2
2	Innova 4100 Axiom Artis BA	6 (LAV, RAV, 2xVCI, RRV, LRV)	2* (LAV, RAV)	15
3	Multistar TOP Artis Zee MP Axiom Artis XA C Polystar XA	3 (LAV, RAV, LFV or RFV)	0	7.5
4	Artis Zee MP	6 (LAV, RAV, VCI, VCS, RRV, LRV)	6 (LAV, RAV, VCI, VCS, RRV, LRV)	7.5

*In case of unclear anatomy, additional DSA was performed.

DSA, digital subtraction angiography; LAV, left adrenal vein; LFV, left femoral vein; LRV, left renal vein; RAV, right adrenal vein; RFV, right femoral vein; RRV, right renal vein; VCI, Vena cava inferior; VCS, Vena cava superior.

### Statistical analysis

Comparison of not normally distributed continuous data was performed using Mann–Whitney *U* test. Normally distributed data are given as mean ± s.d., whereas not normally distributed parameters are shown as median (min–max). Kruskal–Wallis test and *post hoc* analysis (Dunn–Bonferroni) were used to compare DAP and FT grouped by number of performed AVS. *P* value <0.05 was considered statistically significant. SPSS version 24.0 (IBM) was used for statistical analysis.

## Results

### General characteristics of AVS

Overall, 658 procedures were assessed. Due to missing data for ED, DAP and FT, two patients were excluded from the study (*n* = 656). Median age of patients was 53 (16–85) years. Ten patients underwent AVS twice. Two centers (center 3, *n* = 400/400; center 4, *n* = 81/151) used rapid cortisol measurements during AVS to determine correct catheter localization. At center 3, bilateral simultaneous catheterization of adrenal veins was carried out in 35% of cases, whereas all other AVS procedures were performed as sequential catheterization. All AVS were performed without cosyntropin stimulation. Cannulation was successful in 80% of AVS with a large variability across participating centers Table 2. Success rate of AVS was significantly higher when cortisol measurements were performed during AVS (89 vs 56%, *P* < 0.001).

**Table 2:** General characteristics on age, sex, rapid cortisol measurements and success of cannulation. Data are presented as % (*n*) or as mean±S.D.

Center	Age, mean ± s.d.	Females	**Males**	Cortisol measurement	Bilateral simultaneous catheterization % (*n*)	Cosyntropin stimulation	Successful cannulation % (*n*)
1 (*n* = 53)	49 ± 12	45 (24)	55 (29)	No	No	No	59 (31)
2 (*n* = 52)	55 ± 12	58 (30)	42 (22)	No	No	No	61 (31)
3 (*n* = 400)	52 ± 11	64 (256)	36 (144)	Yes	Yes; M: 35 (138)	No	90 (361)
4 (*n* = 151)	57 ± 11	38 (57)	62 (94)	Yes	No	No	F: 50 (35/70)*; M: 82 (66/81)**
Total	53 ± 11	56 (367)	44 (289)				80 (524)

Center 2: *n* = 52 (*n* = 51 for successful cannulation).

*AVS without rapid cortisol measurement; **AVS with rapid cortisol measurement.

### DAP, ED and fluoroscopy time

**Figure 1:**
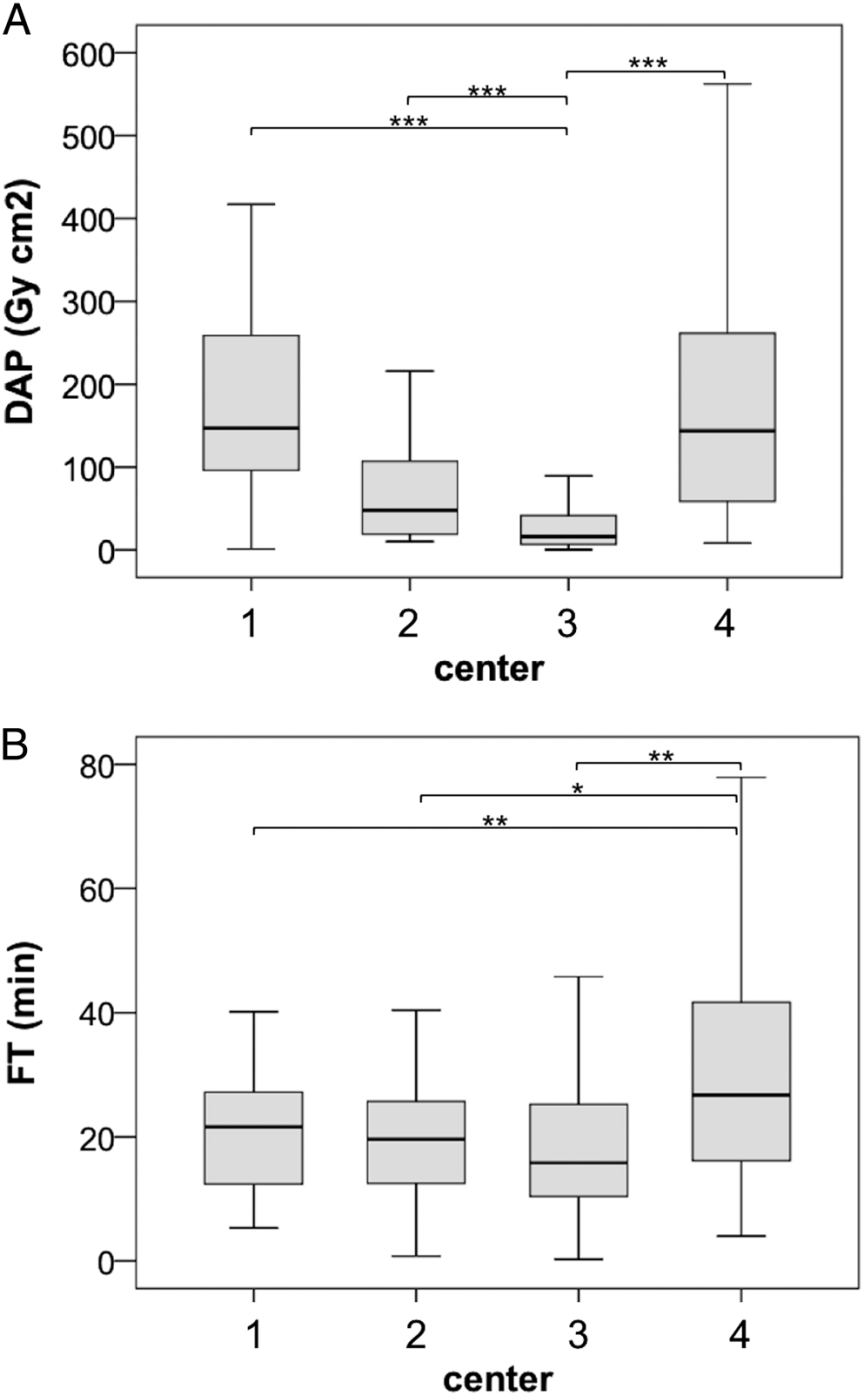
Dose area product (DAP, Gy*cm^2^) and fluoroscopy time (FT, min) in participating centers. (A) DAP, 1 vs 3 *P* < 0.001, 2 vs 3 *P* < 0.001, 3 vs 4 *P* < 0.001, (B) FT, 1 vs 4 *P* = 0.001, 2 vs 4 *P* = 0.002, 3 vs 4 *P* < 0.001. *P ≤ 0.05, **P ≤ 0.01, ***P ≤ 0.001.

Median DAP across all centers was at 33 Gy*cm^2^ (0.3–3181). However, DAP showed high variation among institutions but also within the respective centers, ranging from 16.2 Gy*cm^2^ (0.3–3181) up to 147 Gy*cm^2^ (1.1–1186) Table 3. Especially DAP values recorded at center 3 were significantly lower compared to center 1, 2 and 4 respectively (all *P* < 0.001) Fig. 1. Calculated ED measured 6.5 mSv (0.05–636), again displaying a high variability within and across the centers Table 3. Ten patients underwent AVS twice, leading to a median cumulative ED of 36 mSv (0.92–113.95). Overall, FT was 18 min (0.3–184). FT varied from 16 min (0.3–184) at center 3 up to 27 min (4.0–116) at center 4 (3 vs 4 *P* ≤ 0.001). Table 3 + Fig. 1. Furthermore, radiation exposure significantly increased with age. Whereas for example patients ≤40 years (*n* = 78) received a median ED of 3.4 mSv (0.1–237), patients >70 years (*n* = 44) were exposed to 15 mSv (1.2–178) (*P* < 0.001). Additionally, median ED was significantly lower in female patients (8.9 vs 4.0 mSv, *P* < 0.001).

**Table 3:** Dose area product, effective dose and fluoroscopy time across participating centers. Values are displayed as median (min–max).

Center	DAP (Gy*cm^2^)	ED (mSv)	FT (min)
1	147 (1.1–1186)	29 (0.2–237)	22 (5.3–40)
2	48 (10.0–610)	9.6 (2.0–122)	20 (0.8–50)
3	16 (0.3–3181)	3.2 (0.1–636)	16 (0.3–184)
4	144 (8.2–1166)	29 (1.6–233)	27 (4.0–116)

DAP, dose area product (Gy*cm^2^); ED, effective dose (mSv); FT, fluoroscopy time (min).

**Figure 2:**
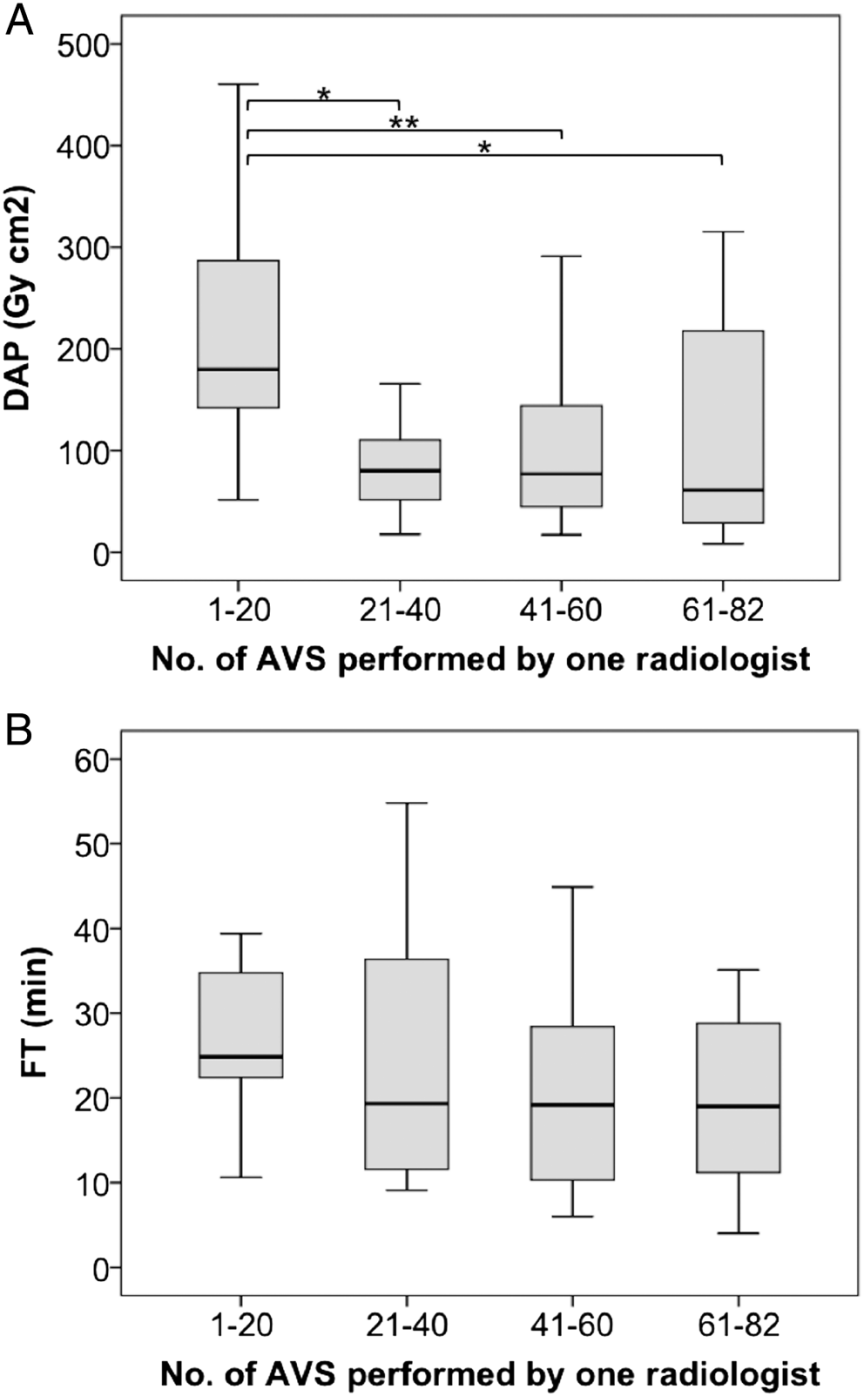
Changes in dose area product (DAP, Gy*cm^2^) and fluoroscopy time (FT, min) over time depending on the number of AVS performed by a single radiologist at center 4 (*n* = 82). *x*-axis = number of AVS in groups of 20 (22) AVS. (A) DAP, 1–20: 180 (52–461); 21–40: 80 (18–339); 41–60: 77 (17–291); 61–82: 61 (8.2–315); 1–20 vs 21–40 *P* = 0.011; 1–20 vs 41–60 *P* = 0.008; 1–20 vs 60–82 *P* = 0.013. (B) FT, 1–20: 25 (11–86); 21–40: 19 (9.1–55); 41–60: 19 (6.0–45); 61–82: 19 (4.0–81). *P ≤ 0.05, **P ≤ 0.01.

Regarding experience level of performing radiologists, radiation exposure decreased with increasing numbers of performed procedures Fig. 2.

**Figure 3:**
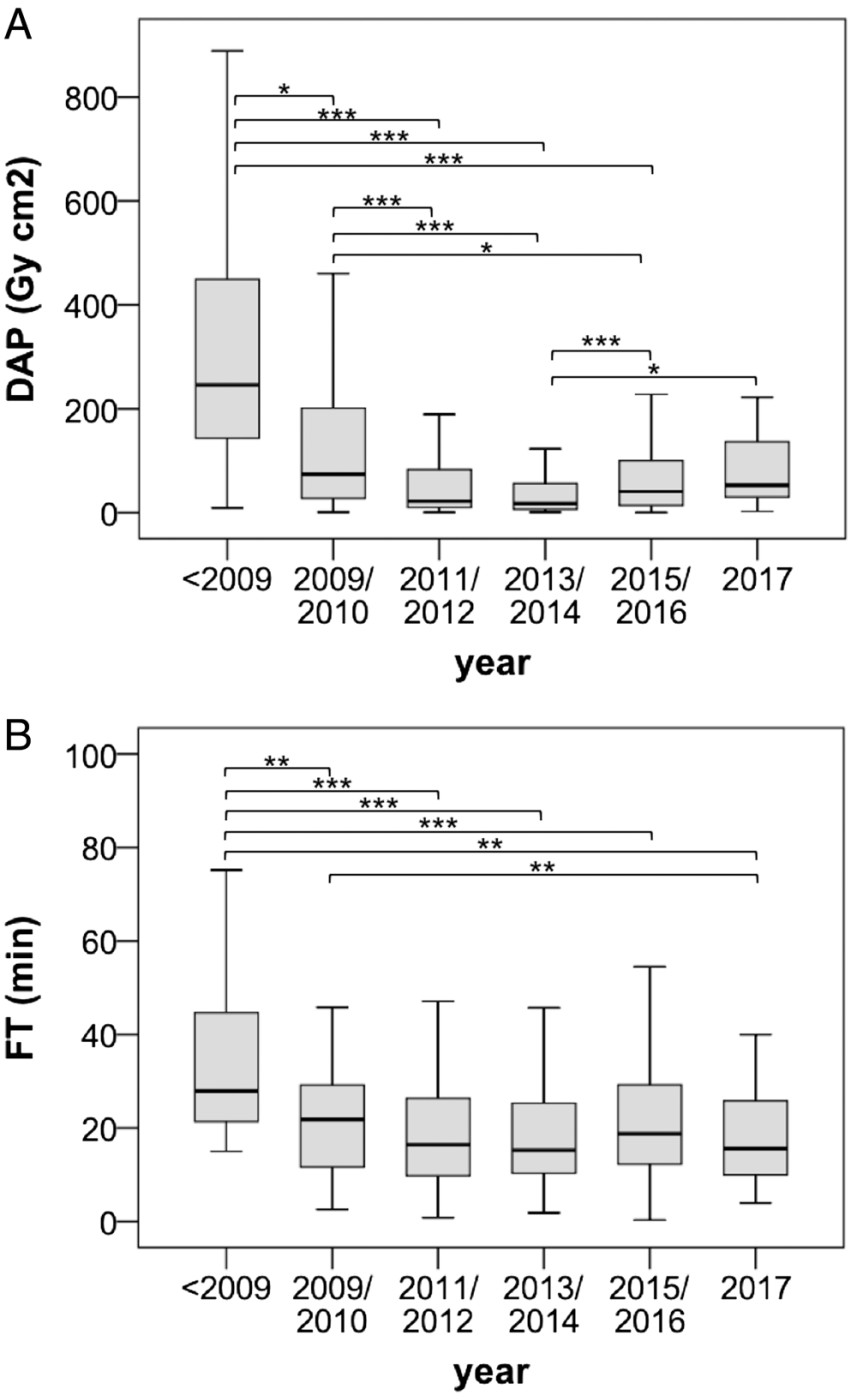
Dose area product ((A), DAP, Gy*cm²) and fluoroscopy time ((B), FT, min) across different years. <2009: *n* = 39, DAP 246 (9.2–888), FT 28 (15–99); 2009/2010: *n* = 75, DAP 74 (1.1–705), FT 22 (2.6–116); 2011/2012: *n* = 119, DAP 22 (0.8–789), FT 16 (0.8–104); 2013/2014: *n* = 177, DAP 18 (1.2–1167), FT 15 (1.9–103); 2015/2016: *n* = 212, DAP 41 (0.3–3181), FT 19 (0.3–184); 2017: *n* = 24, DAP 53 (2.5–222), FT 16 (4.0–63). *P ≤ 0.05, **P ≤ 0.01, ***P ≤ 0.001.

DAP, ED and FT were also significantly lower in successful AVS (FT_successful cannulation_ = 16 min (0.3–184), FT_unsuccessful cannulation_ = 28 min (4.4–116), DAP_successful cannulation_ = 25 Gy*cm^2^ (0.3–3181), DAP_unsuccessful cannulation_ = 80 Gy*cm^2^ (1.1–1186), ED_successful cannulation_ = 5.0 mSv (0.1–636), ED_unsuccessful cannulation_ = 16 mSv (0.2–237), all *P* < 0,001). Over the period of 18 years, there were significant differences in DAP, ED and FT with predominantly consistent values in more recent years. Fig. 3. At center 3 DAP, ED and FT were significantly higher in cases of bilateral simultaneous catheterization compared to AVS with sequential catheterization of adrenal veins (DAP_simultaneous_ = 27 Gy*cm^2^ (0.3–3181), DAP_sequential_ = 14 Gy*cm^2^ (0.8–423), *P* < 0.001; ED_simultaneous_ = 5.4 mSv (0.1–636), ED_sequential_ = 2.8 mSv (0.2–85), *P* < 0.001; FT_simultaneous_ = 18 min (0.3–184), FT_sequential_ = 14 min (1.9–104), *P* = 0.001).

### Sampling locations and imaging modalities

Number of standard sampling locations varied between three (center 3) and six (centers 1, 2 and 4) (Details see Table 1). Digital subtraction angiography (DSA) was performed regularly in three centers (centers 1, 2 and 4) for two, three or all six locations, as well as in cases of unclear anatomy of the adrenal veins. In contrast, sampling locations in center 3 were documented just by using last image hold, but not an additional DSA. The number of frames per second ranged between 7.5 and 15. All centers used low-dose protocols implemented in respective X-ray system and reduced field of view to the necessary minimum. An overview of different imaging modalities is presented in Table 1.

## Discussion

In this study, we demonstrate that AVS is associated with a relevant exposure to radiation in some but not all centers who participated in this study. Even though overall ED measured 6.5 mSv, this was highly variable across participating centers and respective patients (0.1–636 mSv). Highest median ED for one center was at 29 mSv, a value equivalent to 1470 chest X-rays (21) and 12 times the natural background radiation of approximately 2.4 mSv per year (22). For further reference, abdominal CT alone, routinely performed in patients with PA to assess the presence of nodules and for visualization of adrenal veins prior to AVS, is associated with an ED of approximately 10 mSv (23).

It is well known that there are certain risks linked to radiation exposure, such as skin reactions, DNA damage and ultimately cancer induction (24). Whereas skin lesions and cataract are regarded as predictable deterministic effects occurring above a threshold radiation dose estimated at 500 mSv (25), cancer induction represents a stochastic risk, making it nearly impossible to determine radiation as the specific cause of cancer in single patients, especially due to the long latency period between radiation exposure and clinical manifestations. Data available on malignancies in PA showed a lifetime malignancy occurrence of 9.6% in PA patients compared to 6.0% of hypertensive controls which, however, did not reach statistical significance (*P* = 0.08) (26). However, several diagnoses of malignancy had been made prior any AVS in this cohort (26) and long-term data on malignancy occurrence after AVS are so far missing.

Another main result of this study is the difference in radiation doses between participating institutions, which can be explained by the respective variation of imaging modalities. In contrast to all other centers, center 3 performed less samplings in fewer locations and, most importantly, did not use DSA for visualization of vascular structures at any point, but instead documented catheterization of adrenal veins by dynamic fluoroscopic sequences or using last image hold, allowing for the last image to be saved and displayed on the monitor after stopping fluoroscopy. The use of DSA causes approximately ten times higher radiation doses compared to fluoroscopic sequences, therefore being not only a plausible, but also an adjustable factor explaining differences in radiation exposure across participating centers. Furthermore, the use of 7.5, instead of 15 or even 30 frames per second, the patient’s distance from the X-ray tube and the detector, the correct use of collimators and the system settings used for performance of AVS account for more possible influencing parameters. Interestingly, bilateral simultaneous catheterization was associated with a significant increase in radiation exposure.

Based on the results of this study, there is obvious room for improvement regarding technical performance of AVS. To minimize radiation exposure, we recommend adopting some adjustments to currently applied protocols in the future: Documentation of adrenal veins should be done using predominantly last image hold, therefore limiting DSA to a minimum. Additionally, it seems possible to reduce number of sample locations to both adrenal veins and the inferior vena cava without compromising the validity and quality of AVS results, also resulting in lower FT and DAP. The radiation field should be limited to the necessary minimum. These adjustments should not only be considered to reduce patient exposure, but also the operator dose, mainly caused and determined by patient scatter (27). Our data furthermore indicate that the experience of the investigator is of relevance. In addition, centers performing rapid cortisol measurements during AVS showed much higher success rates of >80% in comparison to those without application of this technique (28). Off note, this was not associated with higher EDs to the patients as similarly documented by a recent study (29). To avoid repeated AVS, it may therefore be postulated this procedure is performed in centers with sufficient volumes of investigations and that rapid cortisol measurement becomes standard, aiming at success rates comparable to available data (28, 29). In addition to implementing changes regarding execution of AVS, radiation exposure should generally be kept in mind, when making the decision to perform AVS, even more so in young patients.

There are some limitations to this study: Data on BMI of patients, a factor known to influence DAP, was not recorded (30). However, the vast differences observed between centers are unlikely to be caused predominantly by inhomogeneous distribution of BMI. Regarding variations in DAP, ED and FT between different years, data collection started at different time points at each center (1: 2008, 2: 1999, 3: 2009, 4: 2005). The significantly higher DAP, ED and FT of AVS performed from 1999 to 2008 (*n* = 39) and in 2009 compared to the following years can be attributed both to improved results over time in two centers and mainly by the absence of data from center 3 (<2009: *n* = 0, 2009: *n* = 2), therefore leading to higher values in general. However, it should be noted, that all participating centers were experienced in the performance of AVS.

In conclusion, this first systematic assessment shows that AVS may be associated with fairly high radiation exposure of patients with PA with significant differences across participating centers. We therefore not only suggest taking this risk into account when referring patients to undergo AVS, but also recommend the establishment and application of a common protocol to reduce and prevent unnecessary exposure to radiation.

## Declaration of interest

The authors declare that there is no conflict of interest that could be perceived as prejudicing the impartiality of this study.

## Funding

This work was supported by the Deutsche Forschungsgemeinschaft (DFG) (within the CRC/Transregio 205/1 ‘The Adrenal: Central Relay in Health and Disease’) to S Hahner, M Reincke and M Fassnacht and by the Else Kröner-Fresenius Stiftung in support of the German Conns Registry-Else-Kröner Hyperaldosteronism Registry (2013_A182 and 2015_A171) to Martin Reincke.
